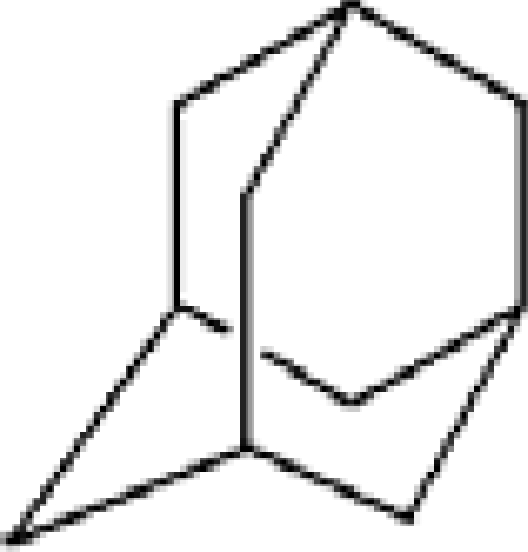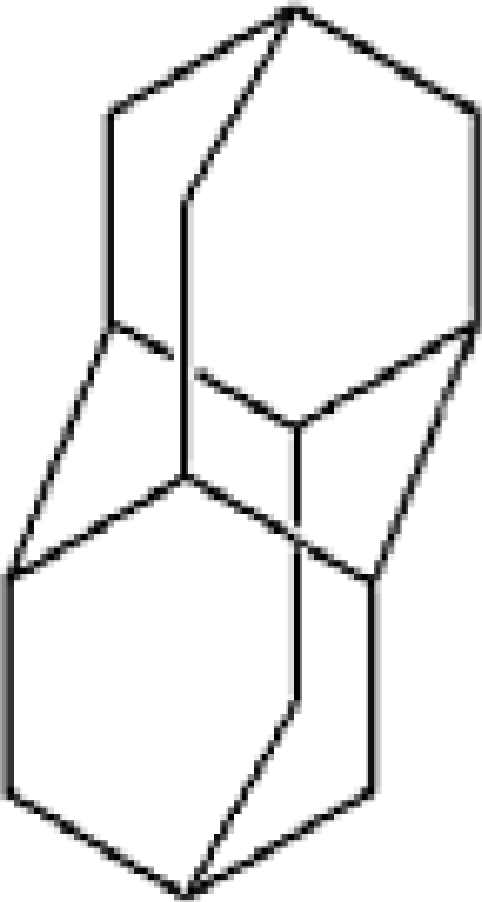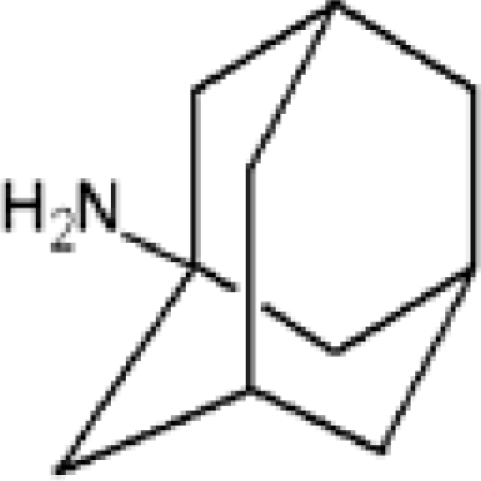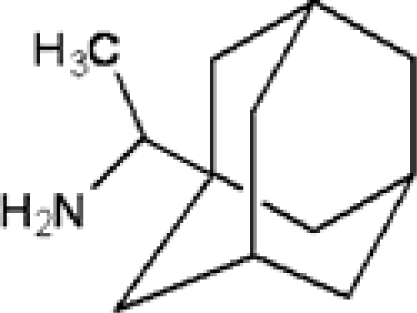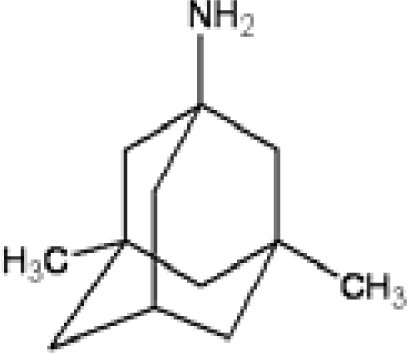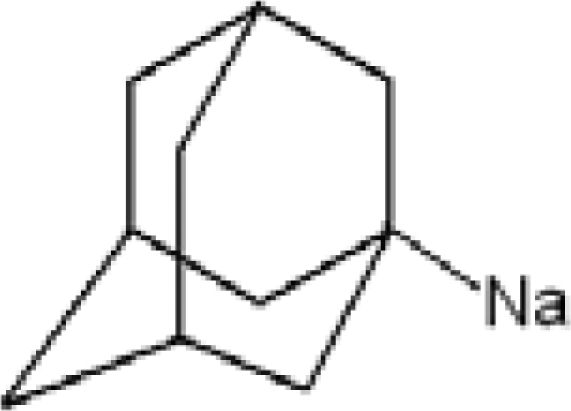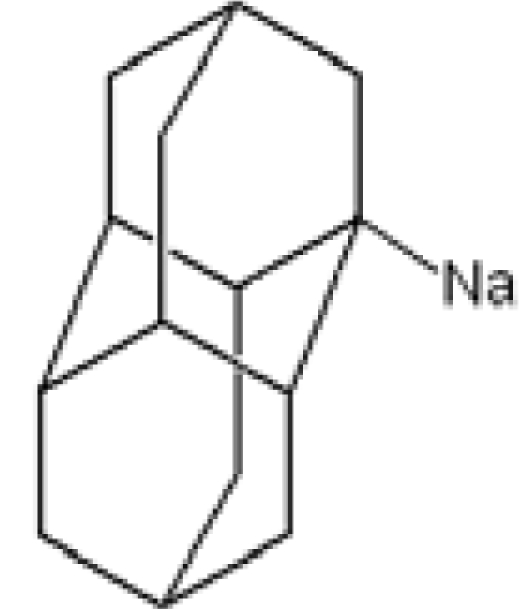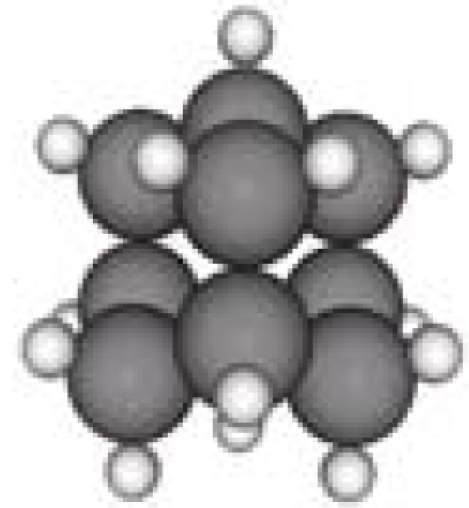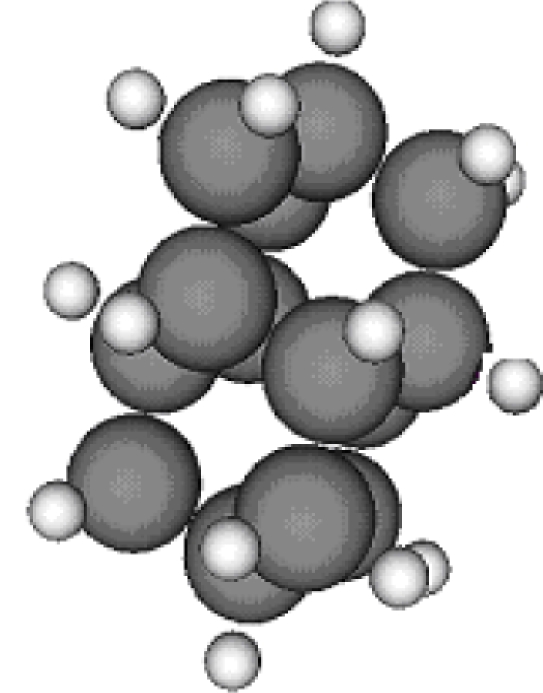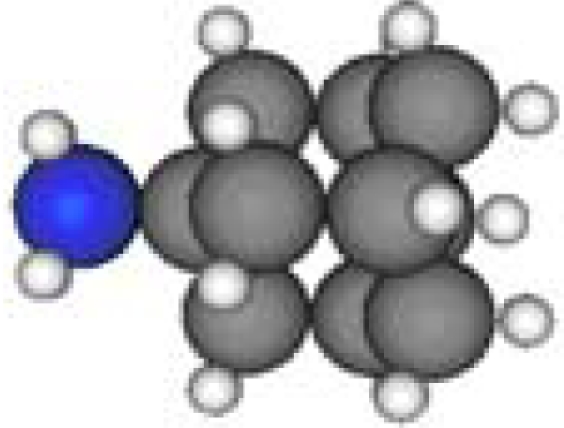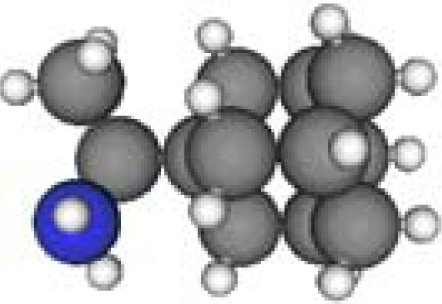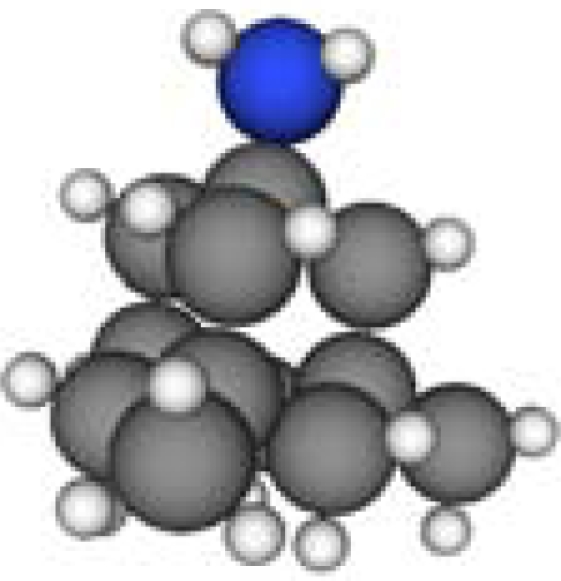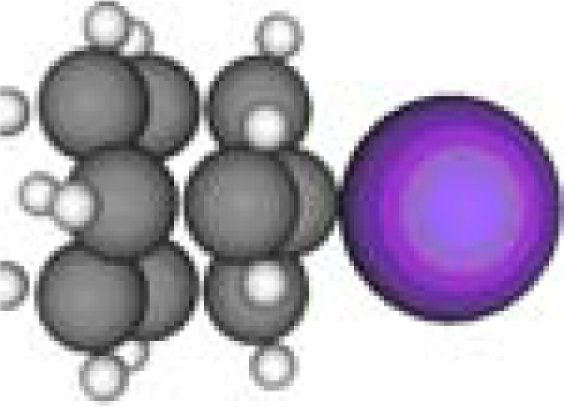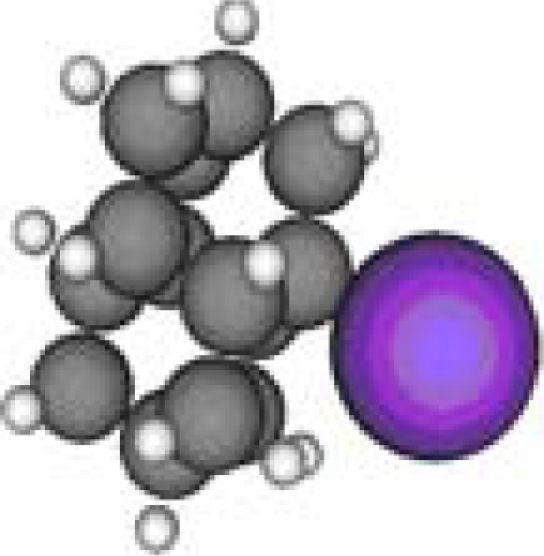# Self-Assembly of Diamondoid Molecules and Derivatives (MD Simulations and DFT Calculations)

**DOI:** 10.3390/ijms11010288

**Published:** 2010-01-21

**Authors:** Yong Xue, G. Ali Mansoori

**Affiliations:** 1 Department of Physics, University of Illinois at Chicago, Chicago, IL 60607-7052, USA; E-Mail: yxue4@uic.edu; 2 Departments of BioEngineering, Chemical Engineering and Physics, University of Illinois at Chicago, Chicago, IL 60607-7052, USA

**Keywords:** adamantane, amantadine, density functional theory, diamantane, diamondoids, MD simulation, memantine, nanotechnology, RDF, rimantadine, self-assembly, simulation annealing, structure factor

## Abstract

We report self-assembly and phase transition behavior of lower diamondoid molecules and their primary derivatives using molecular dynamics (MD) simulation and density functional theory (DFT) calculations. Two lower diamondoids (adamantane and diamantane), three adamantane derivatives (amantadine, memantine and rimantadine) and two artificial molecules (ADM•Na and DIM•Na) are studied separately in 125-molecule simulation systems. We performed DFT calculations to optimize their molecular geometries and obtained atomic electronic charges for the corresponding MD simulation, by which we predicted self-assembly structures and simulation trajectories for the seven different diamondoids and derivatives. Our radial distribution function and structure factor studies showed clear phase transitions and self-assemblies for the seven diamondoids and derivatives.

## 1. Introduction

Diamondoid molecules (which are cage-hydrocarbons) and their derivatives have been recognized as molecular building blocks in nanotechnology [1–10]. They have been drawn more and more researchers’ attentions to their highly symmetrical and strain free structures, controllable nanostructure characteristics, non-toxicity and their applications in producing variety of nanostructure shapes, in molecular manufacturing, in nanotechnology and in MEMS [6,8]. It is important and necessary to study self-assembly of these molecules in order to obtain reference data, such as temperature, pressure, structure factor, bonding properties, *etc*. for application in nanotechnology e.g., building molecular electronic devices.

Two lower diamondoids (adamantane and diamantane), three adamantane derivatives (amantadine, memantine and rimantadine) and two artificial molecules (substituting one hydrogen ion in adamantane and diamantine with one sodium ion: ADM•Na and DIM•Na) are studied in this report. We classified them into three groups, as shown in Table 1.

Group 1: Adamantane and Diamantane, the lowest two diamondoids. Due to their six or more linking groups, have found major applications as templates and as molecular building blocks in nanotechnology, polymer synthesis, drug delivery, drug targeting, DNA-directed assembly, DNA-amino acid nanostructure formation, and host-guest chemistry [1–10]. However these diamondoids do not have good electronic properties which are necessary for building molecular electronics, but some of their derivatives do.

Group 2: Amantadine, Memantine and Rimantadine, the three derivatives of adamantane, have medical applications as antiviral agents and due to their amino groups, they could be treated as molecular semiconductors [8,9].

Group 3: ADM•Na, DIM•Na, the two artificial molecules, substituting one hydrogen ion in adamantane and diamantane with a sodium ion could have potential applications in NEMS and MEMS [8–10]. This report is aimed at studying the self-assembly and phase transition properties of these seven diamondoids and derivatives for further study of their structures and possibly building molecular electronic devices with them.

We first performed density functional theory (DFT) calculations to optimize initial geometry structures of these seven diamondoids and derivatives and obtained their atomic electronic charges [11–16]. Then we performed molecular dynamics (MD) simulation in the study of their self-assembly behaviors. MD simulation methods have been broadly used in studying dynamics of molecules, in spite of its classic approximation [17–21]. As typical plastic crystal, or say molecular crystal, the phase transition behaviors of adamantane molecules have been studied by other researchers using MD simulation method [22,23]. Those studies mainly have focused on the transition of the adamantane from FCC to BCC crystal structure or *vice versa*, and both of these are in the solid phase. Self-assembly, however, is a transition process from a rather random condition of molecules to an ordered state [1]. In the present case of our interest, this process is similar to condensation transition from gas state to liquid state, and then freezing transition from liquid state to solid state, which is a border range of phase transitions. For this reason we chose the Optimized Potentials for Liquid Simulations All Atom (OPLS-AA) force field in our MD simulations. OPLS-AA force field has shown good results not just in the gas and liquid states but also in crystalline phases in a large range of temperatures [17]; moreover, OPLS-AA is a reliable force field for carbohydrates [18] and as a result, obviously, for hydrocarbons. In the OPLS-AA force field, the non-bonded interactions are represented by the general Lennard-Jones plus Columbic form as shown by Equation 1, where *φ_ij_* is the interaction energy of two sites for both intermolecular and intramolecular non-bonded cases:

(1) $$\varphi_{ij}=\sum_{i,j}\left\{4\varepsilon_{ij}\left[{\left(\frac{\sigma_{ij}}{r_{ij}}\right)}^{12}-{\left(\frac{\sigma_{ij}}{r_{ij}}\right)}^{6}\right]+\frac{q_{i}q_{j}e^{2}}{r_{ij}}\right\}k_{ij}$$

The combining rules used along with this equation are *σ_ij_* = (*σ_ii_ σj_j_*)^1/2^ and *ɛ_ij_* = (*ɛ_ii_* *ɛj_j_*)^1/2^. For intermolecular interactions (when *i*, *j* corresponds to different molecules) *k_ij_* = 1.0. While for intramolecular non-bonded interactions between all pairs of atoms (*i* < *j*) separated by three or more bonds, the 1, 4-interactions are scaled down by *k_ij_* = 0.5.

Our MD simulations are mainly performed with the use of the Gromacs package [19–21] and in order to perform simulations involving small molecules, we added to Gromacs the necessary ITFs “included topology files” for the seven different diamondoids and derivatives we studied. Moreover, Gromacs package is capable enough to accept and apply OPLS-AA force field for MD simulations as well as the fact that it is a fast package with flexible characteristics.

We report a brief summary of our MD simulation strategy: vacuum simulation strategy was used in the first step to determine how many molecules were suitable for our simulation and to obtain equilibrated stable structures. Then we built periodic boundary simulation box; and we used the fast Particle-Mesh Ewald (PME) electrostatics method for the preparation of gas state while we chose the cut-off technique for electrostatics in the self-assembly/freezing procedure.

To simulate the self-assembly (*i.e*., freezing transition), we applied the simulation annealing technique to gradually decease the system temperature. V-rescale temperature coupling was applied throughout and in all the simulations. This is a temperature-coupling method using velocity rescaling with a stochastic term [18]. The van der Waals force cut-off and neighbor-searching list distance were, both, set to 2.0 nm, which was large enough since above 1.4 nm the van der Waals force was negligible.

Furthermore, in order to obtain the geometries and atomic charges for the MD simulations of all the seven different diamondoids and derivatives we applied the DFT calculation which is a widely used method in quantum chemistry and electronic structure theory.

## 2. Density Functional Theory (DFT) Calculations

By applying the DFT calculations through Gaussian 03 package [15], we obtained the optimized initial structures of all the seven diamondoids and derivatives and also their atomic electronic charges for the MD simulations. The B3YLP exchange-correlation functional [12] method was chosen with cc-pVDZ basis [14], for all the molecules except for ADM•Na and DIM•Na for which 6-311 + G(d, p) basis [13] was used since sodium was not included in cc-pVDZ basis; NBO (Natural Bond Orbital) analysis [16] was added to calculate the atomic electronic charges which were used as reference to set the atomic charges of nitrogen and sodium atoms, *i.e*., −0.76 and 0.65, respectively (in electronic charge units: Coulomb). The atomic charges of carbon and hydrogen atoms were mainly opted from default OPLS-AA force field.

## 3. Molecular Dynamic (MD) Simulation Procedures and Results

**Figure 1.**
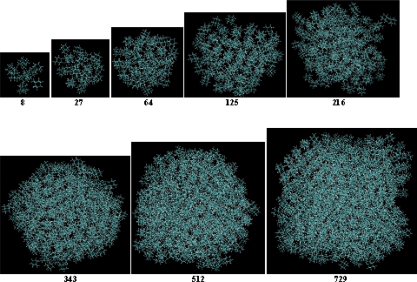
Snapshots from MD simulations using various numbers of adamantine molecules (2^3^, 3^3^, 4^3^, 5^3^, 6^3^, 7^3^, 8^3^, 9^3^) in vacuum with the temperature set at 100 K.

The first step was to determine the number of molecules in the simulation box which was suitable to resemble an NVT ensemble. We performed short (20–40 picoseconds) MD simulations of different numbers of adamantane molecules, (*i.e*., 8, 27, 64, 125, 216, 343, 512, 729 molecules) in vacuum with the temperature set at 100 K, and we successfully obtained all the stable structures as shown in Figure 1.

Although these structures are stable, they may not be used for structural analysis, since the simulations are rather too short. We concluded that a 125-molecule system was big enough to represent the self-assembly behavior of larger systems, which also could save us calculation cost. Therefore, we chose 125 molecules in what is reported in the rest of this paper.

**Figure 2.**
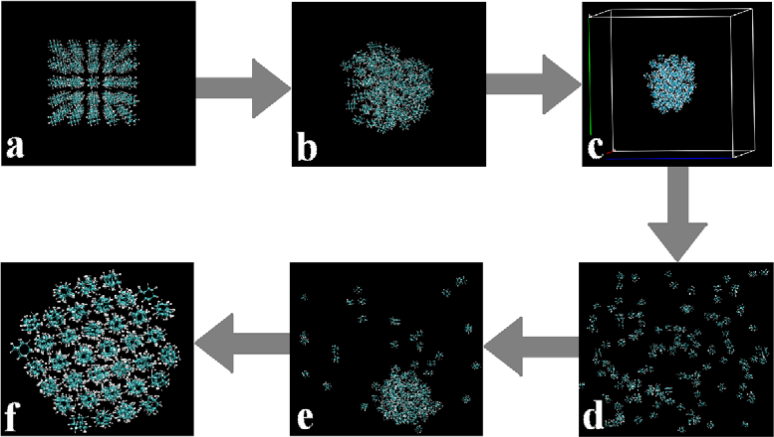
Stages of simulation procedure: (a) Initial 5 × 5 × 5 MD simulation box; (b) Molecules relaxed in vacuum at 100 K after a short MD simulation; (c) Boxed simulation system with overall gas density; (d) Gas phase as a result of equilibrating simulation in the NVT ensemble; (e) The liquid state; (f) Final self-assembly of molecules to solid state.

From this point on, all the MD simulation systems involved 125 molecules for the seven different diamondoids and derivatives. In order to perform self-assembly simulations from gas state to liquid state and eventually solid state, we developed the following procedure for the seven diamondoids and derivatives:

- A MD simulation box with 5 × 5 × 5 = 125 molecules was built by the intrinsic tools in the Gromacs software, as shown in Figure 2a.
- Then we performed a short MD simulation by letting all the molecules relax in a vacuum at 100 K (as in Figure 1) to obtain the stable self-assembled structure, as shown in Figure 2b.
- We boxed those stable structures and set the distances from molecules to the boundaries of the simulation boxes to about +3 nm. In this manner we could build simulation systems with reasonable densities which were in the range of their gas states and also made the systems isolated from the adjacent simulation boxes under the periodic boundary conditions, as shown in Figure 2c.
- Longer (more than 1,000 picoseconds) equilibrating simulations were performed in the NVT ensembles, and we applied the PME method with the cut-off at 2.0 nm and high temperatures (in the range of 500–700 K) in order to make sure the entire system went to the gaseous state, *i.e*., all the molecules separated from each other and distributed randomly in the simulation box as shown in Figure 2d.
  By applying the above four-step procedure we could equilibrate the system in a gas state. As a result, we eliminated the problem due to directly equilibrating the initially prepared molecular system, Figure 2a, at high temperatures. The equilibrated and stable-structure gaseous state, Figure 2d, was then ready for cooling down towards the self-assembly.
- The next step of the simulation was the cooling down the equilibrated and stable-structure gaseous state, Figure 2d, from the gas to the liquid state as shown in Figure 2e.
- Further cooling down of the system resulted in the complete self-assembly of all the molecules (to solid state) as shown in Figure 2f.

**Figure 3.**
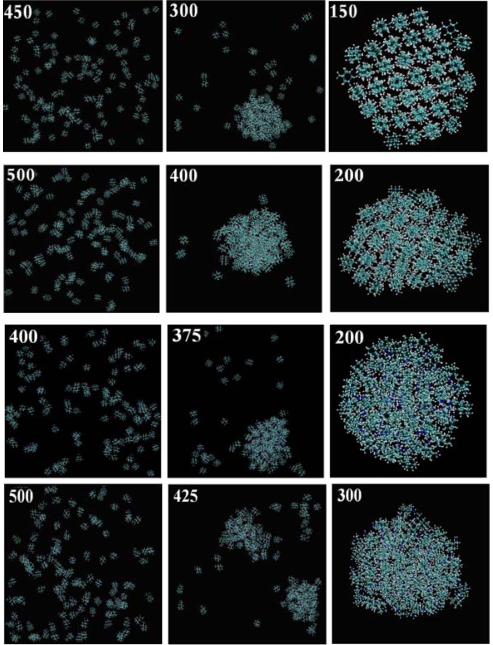

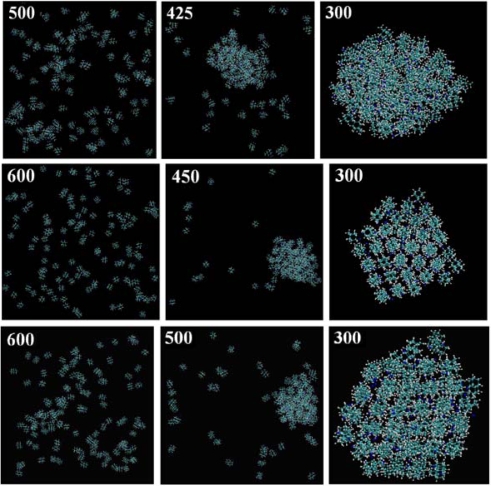
Self-assembly snapshots (left to right) of 125 molecules of the seven diamondoids and derivatives (from top: Adamantane, Diamantane, Amantadine, Rimantadine, Memantine, ADM•Na., DIM•Na) as the temperature [in *K* shown on every snapshot] is decreased.

The snapshots of the gas-liquid-solid MD simulations for 125 molecules (stages d, e and f of Figure 2) of each of the seven diamondoids and derivatives (*Adamantane, Diamantane, Amantadine, Rimantadine, Memantine, ADM•Na*, *DIM•Na*) are reported in Figure 3.

From these snapshots we can directly observe clear phase transitions for each kind of molecules, from the gaseous state to the liquid state, and then their aggregation into a highly condensed (self-assembled) state.

In order to find the equilibrium configuration of the collection of the 125 molecules at every given temperature we used the simulation annealing procedure [1,19]. Every change of 1 K occurred within 10 picoseconds (5,000 time-steps and each time-step was 0.002 ps). With these settings we could observe the self-assembly behavior in the cooling step.

It should be mentioned that we applied the cut-off of 2.0 nm for the electrostatics instead of PME, which was used in step d (Figure 2), in order to: (i). make sure all the interactions among molecules are considered, even beyond 1.4 nm, which is the custom cut-off setting, since after 1.4 nm there is no significant van der waals force; (ii). thus save the computation time at the mean time, since PME requires more computation than the cut-off method.

**Figure 4.**
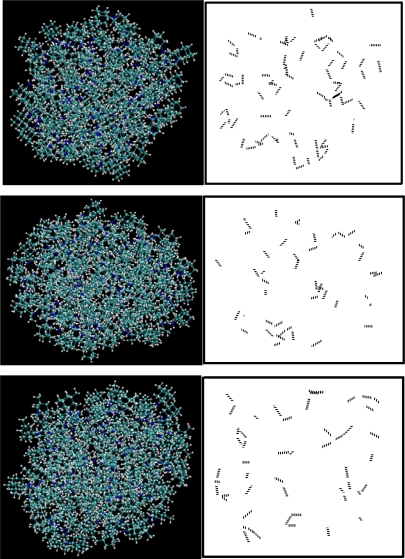
MD snapshots of (from top to bottom) Amantadine, Rimantadine and Memantine at 50 K and their hydrogen bonds locations and orientations.

According to Figure 3 adamantane, diamantane, ADM•Na and DIM•Na form ordered (crystalline) condensed states. However, amantadine, rimantadine and memantine, while they self-assemble, they do not seem to form clear ordered (crystalline) condensed states. We further produced the hydrogen bonds locations of the same self-assembled snapshots of amantadine, memantine and rimantadine as shown in Figure 4.

According to Figure 4 we do not observe any ordered format for the location of hydrogen-bonds, which is an indication of the non-crystalline self-assembled states of amantadine, rimantadine and memantine. Obviously the existence of partial hydrogen-bonds between these molecules is the reason for the lack of a clear crystalline state in their self-assemblies.

We also produced the radial distribution functions (RDFs) and structure factors (SFs) of the seven different diamondoids and derivatives as presented and discussed below. Study of the RDFs and SFs also reveal further about these phase transition features as discussed below. The RDFs we studied and report here are for the centers of the geometry/mass of molecules.

### 3.1. Adamantane and ADM•Na

**Figure 5.**
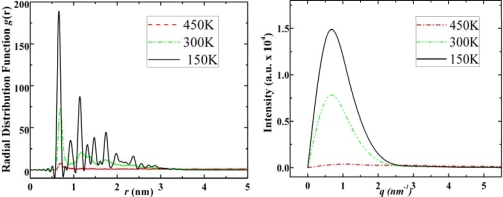
Radial distribution functions (left) and structure factors (right) of adamantane at 450 K, 300 K and 150 K for its gas, liquid and solid states, respectively.

The main feature of adamantane is that at low temperatures we can observe ordered crystal structures (the top-right image in Figures 3), which matches the previous experimental and theoretical studies [22]. From the RDF and SF graphs of adamantane at different temperatures (Figure 5) we can observe gas, liquid and solid characteristics.

**Figure 6.**
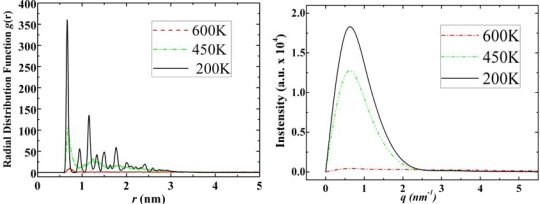
Radial distribution functions (left) and structure factors (right) of ADM•Na at 600 K, 450 K and 200 K for its gas, liquid and solid states, respectively.

In the RDF figures of adamantane, higher and sharper peaks can be observed as the temperature decreases and for the SF graphs, the intensity increases as phase transits from gas to liquid and to solid state. We observed similar features in the RDF and SF figures of of ADM•Na as shown in Figure 6.

The phase transition temperatures for ADM•Na are higher than those of adamantane. However, this is attributed to the presence of -Na ion affecting the molecular interactions among adamantanes, *i.e*., -Na ion in the ADM•Na structure causes stronger bonding than that of the respective -H ion in adamantane. We may also conclude that while the higher phase transition temperatures in ADM•Na, compared to those of adamantane, are due to –Na ion, the geometric structure is determined by the structure of adamantane.

### 3.2. Diamantane and DIM•Na

**Figure 7.**
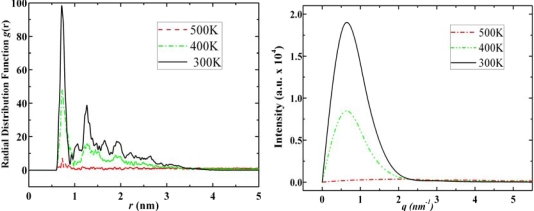
Radial distribution functions (left) and structure factors (right) of diamantane at 500 K, 400 K and 300 K for its gas, liquid and solid states, respectively.

Diamantane molecules can self-assemble to a certain type of solid state structure which can be observed from simulation snapshots (Figure 3), however, its self-assembled structure is not as neat as the crystal structure of adamantane. In Figure 7 we report the RDF and structure factor of diamantane in various phases.

**Figure 8.**
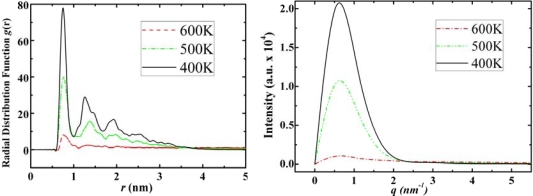
Radial distribution functions (left) and structure factors (right) of DIM•Na at 600 K, 500 K and 400 K for its gas, liquid and solid states, respectively.

From the RDF and structure factor of diamantane, Figure 7, we may also conclude the following: At low temperatures diamantane does not have features of neat crystal structure. DIM•Na shows similar relationship to diamantane as ADM•Na to adamantane, *i.e*., higher phase transition temperatures due to sodium, while similar self-assembled crystal structures as diamantane molecules (See DIM•Na snapshots in Figure 3 and DIM•Na RDFs in and SFs in Figure 8).

### 3.3. Amantadine, Rimantadine and Memantine

These three adamantane derivatives self-assemble at higher temperatures compared with adamantane, but they do not seem to have well-organized self-assembled structures as shown in their snapshots in Figure 4. The reasoning for the special self-assembled structures of amantadine, rimantadine and memantine is as follows:

- . Since there are nitrogen ions in the structure of amantadine, rimantadine and memantine, which makes their attractive intermolecular forces much larger than that of adamantane, higher temperatures should be applied to these systems in order to obtain initial gas state structures in the step d of Figure 2.
- . During the cooling down process of amantadine, rimantadine and memantine, hydrogen-bonds are formed which, as expected, increases their phase transition temperatures compared to that of adamantane.

**Figure 9.**
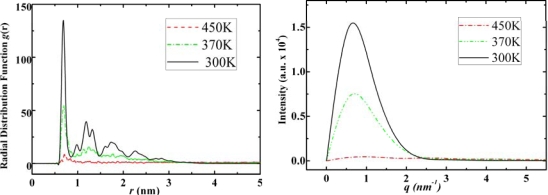
Radial distribution functions (left) and structure factors (right) of amantadine at 450 K, 370 K and 300 K for its gas, liquid and solid states, respectively.

**Figure 10.**
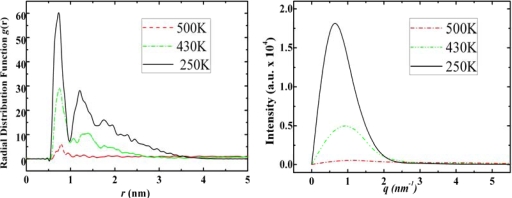
Radial distribution functions (left) and structure factors (right) of rimantadine at 500 K, 430 K and 250 K for its gas, liquid and solid states, respectively.

**Figure 11.**
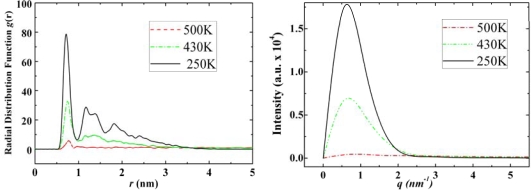
Radial distribution functions (left) and structure factors (right) of Memantine at 500 K, 430 K and 250 K for its gas, liquid and solid states, respectively.

As it is observed from their self-assembled structures (snapshots in Figure 4) amantadine, rimantadine and memantine form certain types of self-assembled structure. However, those structures do not seem to be ordered as was in the case of adamantane, *i.e*., they have no apparent ordered crystalline structure. There may be two main reasons for this: (a). the -NH_2_ and -CH_3_ groups present in these molecules break the geometrical symmetry of adamantane; (b). we observed that the hydrogen bonds, due to -NH_2_ groups are randomly distributed in the bulk structures which makes the entire structures far from an ordered one. In Figures 8–11 we report the RDFs and SFs of amantadine, rimantadine and memantine for their gas, liquid and self-assembled states.

According to Figures 9–11 the self-assembled RDFs and SFs analysis of amantadine, rimantadine and memantine show that for most part there is less obvious ordered self-assembled features, *i.e*., due to lack of sharp peaks as was the case in adamantane. The liquid-state RDF graphs for amantadine, rimantadine and memantine also, for most part, have less obvious liquid-state features either.

**Figure 12.**
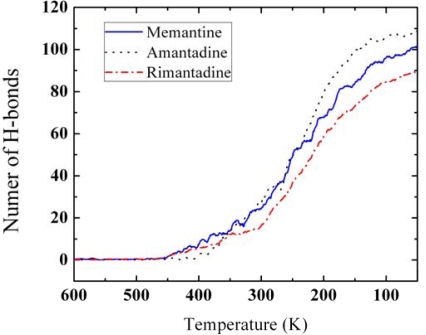
The number of hydrogen bonds for amantadine, memantine and rimantadine vs. temperature. As temperature decreases the numbers of hydrogen-bonds increase, and tend to maximum numbers for the three derivatives.

**Figure 13.**
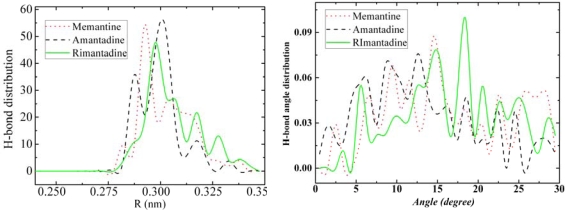
Hydrogen-bond distance distribution (left) and hydrogen-bond angle distribution (right) at 60 K for amantadine, rimantadine and memantine. The most possible hydrogen-bond lengths are around 0.3 nm for the three molecules. The hydrogen-bond angles seem randomly distributed.

We also performed calculations of the hydrogen-bonds saturation as a function of temperature (as shown in Figure 12) as well as hydrogen-bonds distance distribution (as shown in Figure 13-left) and hydrogen-bonds angle distribution (as shown in Figure 13-right) for amantadine, rimantadine and memantine.

According to Figure 12 in the process of phase transition and self-assembly of amantadine, rimantadine and memantine the number of their hydrogen-bonds will reach saturation limit at low temperatures.

From the hydrogen-bond distance-distribution graphs of amantadine, rimantadine and memantine at 60 K (Figure 13-left), we observe several peaks with their highest peaks at about 0.3 nm.

From the hydrogen-bond angle distributions graphs of amantadine, rimantadine and memantine at 60 K (Figure 13-right), we observe that the hydrogen-bond angles are quite randomly distributed for all the three molecules. We already demonstrated the hydrogen-bonds locations and orientations of this group at 50 K (Figure 4) which also showed no orderly features either. Both Figures 4 and 13 indicate that these structures are not orderly self-assembled structures contrary to the adamantane orderly self-assembled state.

In conclusion these three hydrogen-bonded adamantane derivatives just self-assemble at particular temperatures, but may not form well-organized self-assembled crystal structures. The reason is that the geometry structures of these molecules are not symmetric as was the case for adamantane; and their -NH_2_ and -CH_3_ segments, make them unable to pack orderly as adamantane does.

**Figure 14.**
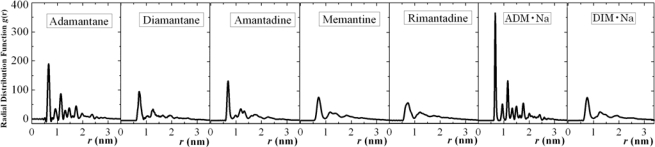
Radial distribution functions of the seven diamondoids and derivatives (from left: Adamantane, Diamantane, Amantadine, Rimantadine, Memantine, ADM•Na, DIM•Na) in the self-assembled (solid) state.

**Figure 15.**
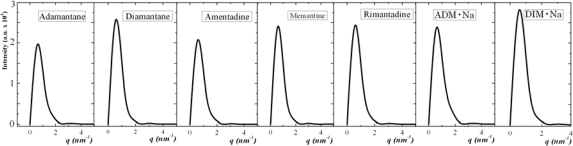
Structure factors of the seven diamondoids and derivatives (from left: Adamantane, Diamantane, Amantadine, Rimantadine, Memantine, ADM•Na., DIM•Na) in the self-assembled (solid) state.

In Figures 14 and 15 we report the self-assembled (solid-state) radial distribution functions and structure factors, respectively, of all the seven diamondoids and derivatives with uniform coordinates scales for the purpose of their collective comparison.

According to Figures 14 and 15 we can observe that adamantane and ADM•Na have more sharp peaks than the other five, which is the indication of their orderly self-assembled structures. While other five molecules also show self-assembled characteristic peaks they have less number of such sharp peaks than adamantane and ADM•Na. These results match the image observation of simulations (Figure 3), *i.e*., adamantane and ADM•Na have more orderly structures in their self-assembled states due to their molecular symmetry, which thus proves that self-assembly of those molecules are structure-dependent.

## 4. Conclusion

We have performed a detailed molecular dynamics study of the self-assembly process of seven different diamondoids and derivatives due to temperature variations. From the MD simulation study of the seven diamondoids and derivatives, we may conclude the following: (1) The nature of self-assembly in these molecules is a structure-dependent phenomenon. (2) Final self-assembly structures depend on the different bonding types present in the molecular and intramolecular interactions of these various molecules. (3) The artificial molecules (ADM•Na., DIM•Na) still hold neat crystal structures. Although -Na ion increases the phase transition temperature, as does -NH_2_ in amantadine, rimantadine and memantine. (4) To a large extent the structural features of diamondoids are retained in ADM•Na and DIM•Na. The reasons for the latter might be that: A. The -Na ion has less topology effect than does the -NH_2_. B. There is no hydrogen-bonding in the structures of ADM•Na and DIM•Na; therefore they can aggregate to ordered self-assembled structures. This feature is very promising, since it allows us to build orderly-shaped self-assemblies suitable for NEMS and MEMS.

## Figures and Tables

**Table 1.** Molecular formulas and 3-D structures of Adamantane, Diamantane, Memantine, Rimantadine, Amantadine, Optimized ADM•Na and Optimized DIM•Na molecules. In these figures black spheres represent “–C”, whites represent “–H”, Blues represent “–N” and purples represent “–Na”.

**Group 1**		**Group 2**			**Group 3**	
**Adamantane**	**Diamantane**	**Amantadine**	**Rimantadine**	**Memantine**	**Optimized ADM•Na**	**Optimized DIM•Na**
***C_10_H_16_***	***C_14_H_20_***	***C_10_H_17_N***	***C_11_H_20_N***	***C_12_H_21_N***	***C_10_H_15_Na***	***C_14_H_19_Na***